# Intermetallics Formation during Solidification of Al-Si-Cu-Mg Cast Alloys

**DOI:** 10.3390/ma15041335

**Published:** 2022-02-11

**Authors:** Adel M. A. Mohamed, Ehab Samuel, Yasser Zedan, Agnes M. Samuel, Herbert W. Doty, Fawzy H. Samuel

**Affiliations:** 1 Metallurgical and Materials Engineering Department, Faculty of Petroleum and Mining Engineering, Suez University, Suez 43721, Egypt; adel.mohamed25@yahoo.com; 2 Département des Sciences Appliquées, Université du Québec à Chicoutimi, Saguenay, QC G7H 2B1, Canada; gen13es@hotmail.com (E.S.); agnesmsamuel@gmail.com (A.M.S.); fawzy-hosny.samuel@etsmtl.ca (F.H.S.); 3 École de Technologie Supérieure, Département de Génie Mécanique, Montréal, QC H3C 1K3, Canada; 4 General Motors Global Technology Center, Materials Technology, Estes Bldg, 30003 Fisher Brothers Rd., Warren, MI 48093-2350, USA; herb.doty@gm.com

**Keywords:** Al-Si alloys, intermetallics, solidification, phase identification, EPMA, porosity, mechanical properties

## Abstract

The present study was undertaken to examine the effect of iron, manganese, copper and magnesium on the microstructural characteristics of Al-11%Si-2%Cu-Mg-based alloy referred to as 396 under different working conditions. The results show that strontium (Sr) has high affinity to react with magnesium (Mg), resulting in reduced effectiveness as eutectic silicon modifier or age hardening agent. In addition, Sr alters the sequence of the precipitation of the α-AlFeMnSi phase from post-eutectic to pro-eutectic which would harden the soft α-Aluminum matrix. The mechanism is still under investigation. The interactions between iron (Fe) and Mg and Sr-Mg result in the formation of serval dissolvable intermetallics during the solutionizing treatment such as β-AlFeSi, π-AlFeMgSi and Q-AlMgSiCu phases. The study also emphasizes the role of modification and grain refining as well as intermetallics in porosity formation and hardness of samples aged in the temperature range 155–240 °C.

## 1. Introduction

It has been proposed that increased solidification rates, strontium addition and the presence of transition elements such as Mn will promote the development of a more compact, less harmful α-Fe phase [1,2,3,4,5,6]. The composition of the α-Fe phase is Al_8_Fe_2_Si (31.6% Fe, 7.8% Si), often reported as Al_15_Fe_3_Si_2_ (30.7% Fe, 10.2% Si), with a probable composition range of 30 to 33% Fe and 6 to ~12% Si. The phase is reported to have a hexagonal structure with the parameters a = 12.3 Å, c = 26.3 Å and a density of 3.58 g/cm^3^; it appears in the form of Chinese-script particles. The α-Fe phase exerts a less deleterious effect on the physical properties of the cast part due to its more compact shape and a more diffuse interface with the aluminum matrix, resulting in better cohesion [7,8]. 

The growth rate of the α-Fe phase occurs at a high degree of undercooling, ΔT, whereas the β-Fe phase (Al_5_FeSi) grows in a lateral or faceted mode which is poorly bonded to the aluminum matrix and contains multiple (001) growth twins parallel to the growth direction. This type of growth occurs at low driving forces or at slow cooling, i.e., at a low degree of undercooling, ΔT [9].

The separation of high-melting-point intermetallic compounds from liquid metal may occur by means of precipitation and gravity segregation. These intermetallics are, in actual fact, solid solutions which have a substantial capacity for dissolving other elements. Although expressed by definite chemical formulae, these compounds exhibit a wide range of compositions and precipitation temperatures [10]. 

According to Cáceres et al. [11], an increased Si content in Al-Si-Cu-Mg alloys leads to refined size of the β-Al_5_FeSi platelets; this may be ascribed to the tendency in the alloys containing high levels of Si to form large particles of pre-eutectic β-Al_5_FeSi and α-Al_15_(Mn,Fe)_3_Si_2_ particles during solidification, as a result of a reduction in the available growth period. The size-refining effect of a high Si content is also evident in other intermetallics such as α-Al_15_(Mn,Fe)_3_Si_2_ and θ-Al_2_Cu which form from the eutectic liquid in Al-Si-Cu-Mg alloys. Thus, the evidence suggests that an increased Si content tends not only to refine the size of intermetallic particles but also to redistribute them into a more uniform dispersion within the intermetallic and intergranular regions compared with a lesser Si content which promotes long clusters of intertwined particles along the grain boundaries. 

The growth and propagation of microcracks nucleated by the cracking of the intermetallics is therefore more difficult and involves greater local plasticity when the particles are further dispersed, increasing the tensile ductility for high Si content alloys. It is known that the degree of Si content may change the primary aluminum grain structure radically, from a globular morphology at Si contents of less than ~6%, to an orthogonal dendritic structure at higher Si levels [12]. It has been suggested that these Si-induced morphological changes in the structure are responsible for the refining effect on the Cu- and Fe-rich intermetallic phases during solidification, in turn leading to the increase observed in tensile ductility [13,14]. 

The focus of this study was to investigate the influence of iron (0.5–1 wt.%), Mn (0.5–1 wt.%), Cu (2.25–3.25 wt.%) and Mg (0.3–0.5 wt.%), on the microstructure of modified and grain-refined previously studied Al-11%Si-2%Cu-Mg alloy (registered as 396) in both as-cast and heat-treated conditions (solution treatment and aging conditions), on the microstructural characteristics and precipitation of intermetallic phases in as-cast near-eutectic Al-Si alloys, with emphasis on the changes occurring in the microstructure as a result of solution heat treatment.

## 2. Experimental Procedure

The Al-11%Si-2%Cu-Mg base alloy used in this research, registered as 396 alloy and referred to as R [15], was received from the supplier in the form of 12.5 kg ingots. Using this base alloy, four main groups of alloys were prepared, corresponding to Sr and Ti, Fe and Mn, Cu and Mg and Pb, Sn and Bi additions, and referred to as R, RF, RC and RT, respectively. The bulk of the experimental work was carried out using the Al-10.8%Si near-eutectic alloy. The as-received Al-10.8%Si ingots were cut into smaller pieces, cleaned, dried and melted in charges of 34 kg each to prepare the required alloys. The melting process was carried out in a SiC crucible of 40 kg capacity, using an electrical resistance furnace. The melting temperature was maintained at 750 ± 5 °C. All alloys were grain-refined by adding 0.25%Ti as Al-5%Ti-1%B in rod form and modified by adding Sr, in the form of an Al-10%Sr master alloy (150 ppm Sr), using a perforated graphite bell. Taking the grain-refined and modified alloys, referred to as R, RM and RGM base alloys, addition of Fe, Mn, Cu and Mg to the RGM alloy was then carried out in order to study the effects of these alloying elements on the microstructure and mechanical properties of the grain-refined and modified alloy. 

All melts were degassed using pure, dry argon injected into the melt for ~15 min by means of a rotating graphite degassing impeller, at 125 rpm rotation, to ensure homogeneous mixing of the additives, and a melt hydrogen level of 0.1 mL/100g. The humidity varied between 11 and 15% when preparing these melts. The degassed melt was carefully poured into various preheated molds to prepare castings for obtaining samples for metallographic observations, hardness measurements, tensile testing and impact testing. The pouring temperature was 730 ± 5 °C. Iron and Mn were added in the form of Al-25%Fe and Al-25%Mn master alloys, respectively, whereas Cu and Mg were added in the form of the pure metal. This produced the alloys classified as groups RF and RC in Table 1. The humidity in the laboratory was about 13% (dry). Prior to casting, samples were tested for porosity using reduced pressure testing apparatus. 

**Figure 1:**
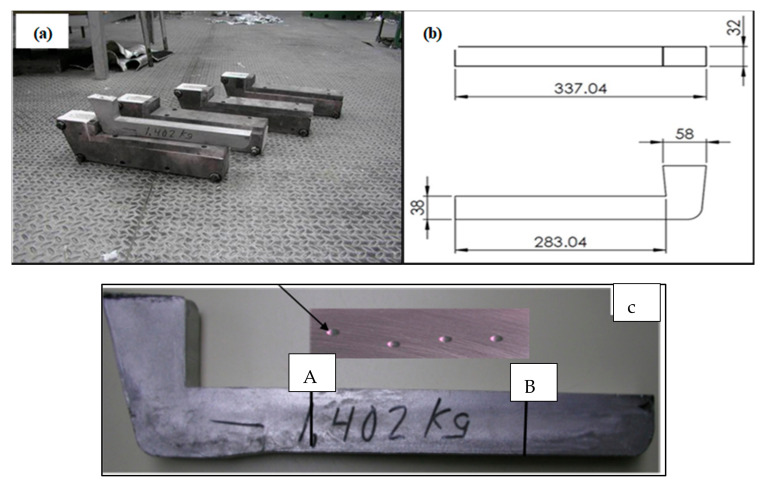
(**a**) Casting used to prepare the metallographic and hardness samples; (**b**) sample drawing and dimensions; (**c**) alloy casting and hardness test bar (2.54 × 2.54 × 7.62 cm) obtained from the casting.

Samples for metallography and hardness testing purposes were obtained from castings produced from the L-shaped metallic mold (preheated at 350 °C prior to casting), as shown in Figure 1a,b. The points A and B in Figure 1c show the location on the casting from which the hardness test bar was sectioned, while the inset shows the surface of such a bar with indentations, resulting from the hardness measurements.

For all alloys, 35 bars were prepared for each alloy composition. The test bars were divided into seven sets: One set was kept in the as-cast condition, while the other six sets were solution heat-treated at 495 °C for 8 h (SHT), then quenched in warm water at 65 °C, followed by artificial aging at 155, 180, 200, 220 and 240 °C for 5 h (i.e., T6- and T7-tempered). Heat treatment was carried out in an air-forced electrical furnace (±2 °C). The hardness measurements were carried out on the as-cast and heat-treated samples using a Brinell hardness tester, employing a steel ball of 10 mm diameter and a load of 500 kgf applied for 30 s. An average of eight readings obtained from two perpendicular surfaces was taken to represent the hardness value in each case.

Samples for metallographic examination were mounted and then subjected to grinding and polishing procedures to produce a mirror-like surface. The mounting of the samples in bakelite was carried out using a Struers Labopress-3 machine, while the grinding and polishing procedures were carried out using a TegraForce-5 machine. The grinding procedures were applied using silicon carbide (SiC) papers having the size sequence of 120, 240, 320, 400, 600, 800 and finally 1200 grit; it should be noted that the word grit is used to represent a measure of fineness for abrasive materials and that water was used as a lubricant in this stage. 

Polishing was carried out using Struers diamond suspension, which contains a diamond particle size of 6 µm, as the first step of the polishing process followed by further polishing through the application of the same suspension containing a smaller diamond particle size of 3 µm. The lubricant used for this polishing stage is a Struers DP-lubricant. The final stage of polishing was carried out using a Mastermet colloidal silica suspension, having a particle size of 0.6 µm. Water was used as lubricant throughout the final polishing stage, after which the samples displayed a mirror-like surface and were ready for microstructural examination. 

Microstructures of the polished sample surfaces were examined using an Olympus PMG3 optical microscope. The eutectic silicon particle characteristics, including area, length, aspect ratio, roundness and density, were measured and quantified using a Clemex image analyzer system in conjunction with the PMG3 optical microscope. For each sample, 50 fields at a magnification of 500× were examined, so as to cover the entire sample surface in a regular and systematic manner. In addition, porosity measurements were carried out, over 30 fields per sample, at a magnification of 50×. The porosity parameters measured were percentage porosity, pore area and pore length. As a rule, the outer edges of a sample were avoided in taking these measurements, to eliminate any distortions that might occur in the peripheral regions. Phase identification was carried out using electron probe microanalysis (EPMA) coupled with energy dispersive X-ray (EDX) and wavelength dispersive spectroscopic (WDS) analyses, using a JEOL JXA-8900l WD/ED combined microanalyzer operating at 20 KV and 30 nA, where the electron beam size was ~2 µm. Mapping of certain specific areas of the polished sample surfaces was also carried out where required, to show the distribution of trace elements in the phases. 

A Hitachi-SU8000 field-emission scanning electron microscope (FESEM), as was used in this study, can provide clear and less electrostatically distorted high resolution images even at low voltages, with an image resolution of 2.1 nm at 1 kV and 1.5 nm at 15 kV. The FESEM instrument also comes equipped with a standard secondary electron detector (SE), a backscatter electron detector (BSE) and an energy dispersive X-ray spectrometer (EDS). 

## 3. Results and Discussion

### 3.1. Grain Refining and Modification

Grain refinement and modification are commonly employed in producing aluminum castings in order to improve their mechanical properties. Aluminum-silicon alloys without modification treatment are characterized by relatively poor mechanical properties due to the presence of coarse acicular plates of eutectic silicon which act as internal stress raisers. Grain structure is also an important feature in aluminum alloy castings. Researchers have often noted that fine grain size is beneficial to castings since feeding characteristics, tear resistance and mechanical properties are all observed to be improved by it. For this reason, grain refiners are often added to the melt before casting so as to obtain a fine equiaxed solidification structure. The addition of such alloying elements as copper and magnesium enhances the mechanical properties of aluminum-silicon casting alloys. In the light of the above, the effects of the addition of melt treatment and alloying elements on the microstructure, hardness and tensile properties of the 396 alloy were studied. According to Tahiri et al. [16,17] increasing the added grain refiner beyond 0.14% Ti using Al- 5%Ti-1%B master alloy would lead to an increase in both grain size, and the amount of decomposed Al_3_Ti (or more precisely (Al,Si)_3_Ti platelets) would result in marked deterioration in the alloy mechanical properties. 

**Figure 2:**
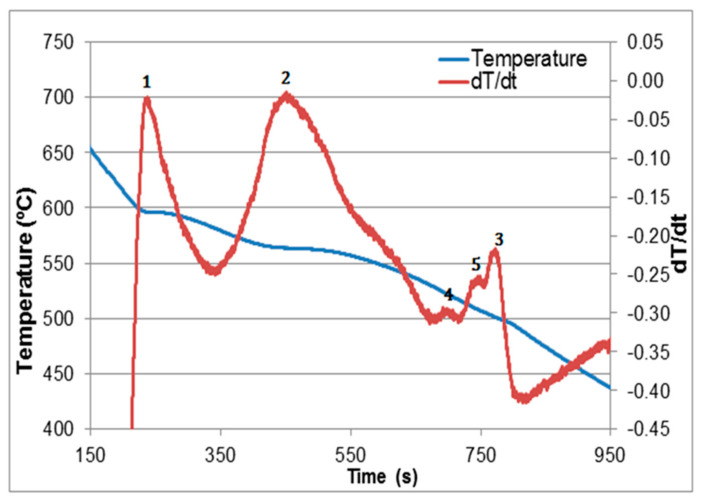
Solidification curve and its first derivative of the base alloy R (0.8 °C/s).

**Figure 3:**
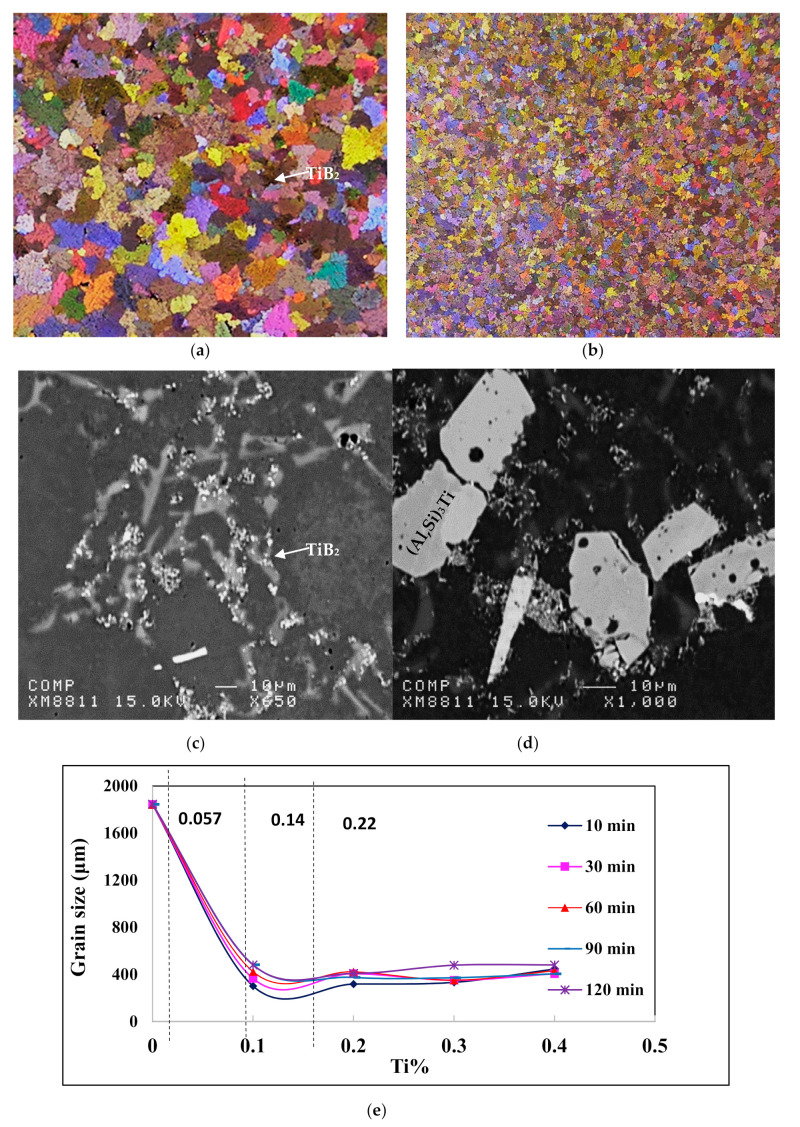
(**a**,**b**) Macrostructure of grains in alloys R and RGM, respectively, (**c**) TiB_2_ distribution in alloy R, (**d**) decomposition of the grain refiner into TiB_2_ and (Al,Si)_3_Ti in alloy RGM, (**e**) effect of grain refiner on the alloy grain size [16].

Figure 2 and Table 2 show, respectively, the solidification curve obtained for alloy R at a rate of 0.8 °C/s, whereas Table 2 lists the possible reactions according to Backerud et al. [10]. The effect of grain refining on the size and distribution of the grains in alloys R and RGM is illustrated in Figure 3. Although increasing the amount of added grain refiner from 0.057%Ti in alloy R to 0.22% in alloy RGM noticeably reduced the grain size, the increase in added Al-5%Ti-1%B to alloy RGM resulted in the precipitation of massive platelets of (Al,Si)_3_Ti which would have a negative effect on the alloy mechanical properties. It is inferred from the work of Tahiri et al. [16] that the appropriate amount of added grain size should be about 0.14% (almost halfway between Ti in alloy R and alloy RGM as shown in Figure 3e). 

**Figure 4:**
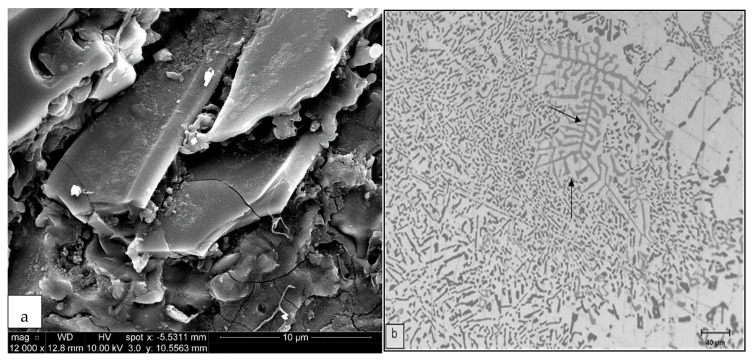
Effect of solution treatment at 495 °C on Si morphology in the base R alloy: (**a**) 0 h, backscattered electron image and (**b**) 8 h, optical micrograph. Arrows point to α-Fe script particles.

The morphology of eutectic Si plays a vital role in determining the mechanical properties of Al-Si alloys. Particle size, shape and spacing are all factors which characterize the structure of silicon. As may be seen in Figure 4a, the Si particles are present in the form of coarse acicular plates with an aspect ratio of 2.41 in the as-cast condition for the base alloy (R). The silicon represents the hard phase of the alloy, which causes a discontinuity of the soft and ductile matrix of aluminum. Because α-Al is the softer phase and Si is the harder and less ductile one, stresses cause anisotropic distribution of the plastic deformation, which is greater in the softer phase. The local plastic constraint in the softer phase leads to a rapid strengthening of the alloy, with dislocations piling up at the α-Al/Si interfaces. This can lead to the formation of cleavage microcracks at these ductile-brittle sites. On such a basis, it is to be expected that differences between the mechanical properties of the five tensile bars will be higher for the unmodified alloy. 

**Figure 5:**
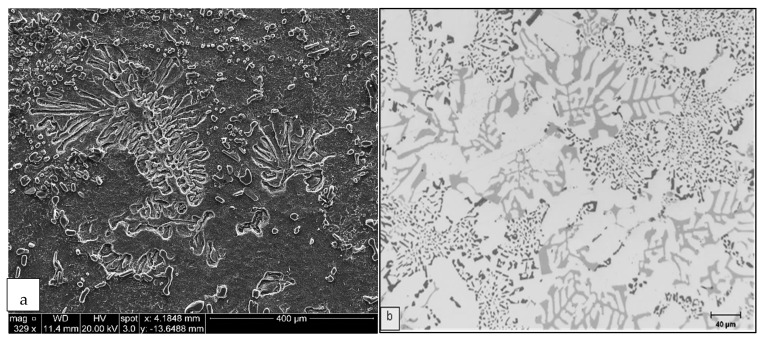
Effect of solution treatment at 495 °C on Si morphology in Sr-modified RM alloy: (**a**) 0 h, backscattered electron image and (**b**) 8 h, optical micrograph.

The addition of 150 ppm of Sr transforms the morphology of the Si particles from an acicular form (R alloy) to a fibrous one (RM alloy). The aspect ratio decreased by 18%, while the roundness ratio increased from 57% to 74%, (see Figure 5a). Correspondingly, the Si particle size, i.e., the average particle length and area decreased by 59% and 72%, respectively. As a result of the decrease in the size of the particles, the density of the Si particles increased by about 350% (from 10,096 to 45,490 particles/mm^2^) implying that in the presence of Sr, the eutectic Si phase was fibrous and finely divided, as indicated in the micrograph shown in Figure 5b. 

The modified structure is often improperly called globular [16,17] since the fibers appear to be small individual globules (particles) on a conventionally polished surface; they are, in fact, connected in a coral or seaweed-like structure. Figure 4a and Figure 5a clearly show the microstructural differences between the unmodified and modified alloys, respectively. In the unmodified alloy, the Si phase was to be observed in the form of large plates with sharp sides and ends known as acicular silicon. The globules of the modified structure are the ends of silicon fibers which form an interconnected network. Such a structural transformation from acicular to fibrous silicon is responsible for the improvement in the mechanical properties of modified castings.

**Figure 6:**
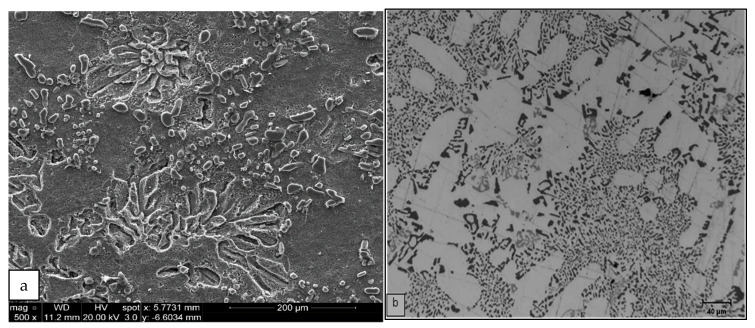
Effect of solution treatment at 495 °C on Si morphology in Sr-modified and grain-refined RGM alloy: (**a**) 0 h, backscattered electron image and (**b**) 8 h, optical micrograph.

The micrograph shown in Figure 6a illustrates the combined effects of Sr and Ti addition on Si particles in the RGM alloy. The primary α-Al phase has a fully columnar (dendritic) structure in the untreated alloy (see Figure 4a) but transforms to an equiaxed morphology with the combined addition, as shown in Figure 6a. Such an effect is believed to be due solely to the grain refiner segment of the combined treatment. The average Si particle area, length and aspect ratio of the RGM alloy in the as-cast condition decreased by 69%, 54% and 12%, respectively, compared to the R alloy, whereas the average roundness and density increased by 24% and 304%, respectively. It should be noted that the average Si particle area, length and aspect ratio of the RGM alloy in the as-cast condition increased by 12%, 11% and 7%, respectively, compared to the RM alloy, whereas the average roundness and density decreased by 3% and 10%, respectively. It may thus be concluded that the addition of Ti has a slight poisoning effect on the effectiveness of Sr addition as a modifier.

Under normal cooling conditions, eutectic silicon forms a network of interconnected irregular flakes. As was observed earlier, the eutectic Si may be chemically modified to a fine fibrous structure. High temperature treatments can also alter Si particle characteristics. In recent years, both chemical and thermal modification have been used in conjunction to produce the desired properties of the casting. Several investigators have used quantitative metallographic techniques to monitor the changes in Si particle morphology during solution heat treatment [18,19]. One of the objectives of solution heat treatment is to allow the soluble hardening elements of the alloy to dissolve into solid solution and to homogenize castings. Because solubility and diffusion rate both increase with temperature, it is usually desirable to use the highest treatment temperature possible without causing melting. When castings are heat treated at temperatures lower than the normal range, dissolution is incomplete, and mechanical properties are not optimum.

The energy state, i.e., the surface curvature and lattice deformation of the discontinuous eutectic phase, is inhomogeneous. During high temperature treatment, a mass transport of the solute occurs from areas of high energy. The silicon atoms in the matrix at such locations diffuse to a lower energy location, resulting in the dissolution of eutectic Si at the former, and the precipitation of Si on the eutectic Si at the latter location, as seen in Figure 6. This mass transport of silicon causes the fragmentation and spheroidization of eutectic silicon, both of which are dependent on the diffusion of both solute atoms and matrix atoms. 

The microstructural changes occurring during solution heat treatment of the as-received Al-11% Si-2%Cu-Mg alloy (R) are shown in Figure 4b. Initially, the Si particles break down into smaller fragments and become gradually coarsened. From Figure 5b and Figure 6b, it may be observed clearly that modification has a profound influence on spheroidization. In the modified alloys (RM and RGM), a high degree of spheroidization followed by coarsening occurs during solutionizing at 495 °C. The microstructural changes resulting from solution heat treatment originate from the instability of the interface between two phases. Plate-like eutectics are more resistant to interfacial instabilities and subsequent spheroidization than the fibrous kind [20]. Thus, the rate of spheroidization is extremely rapid in modified alloys.

Spheroidization and coarsening of the discontinuous phase occurs at elevated temperatures [21] because the interfacial energy of a system decreases with the reduction in interfacial surface area per unit volume of the discontinuous phase. The reduction in interfacial energy is the driving force for the spheroidization and the coarsening processes which are also diffusion-controlled [22,23,24]. The degree of interconnection of the Si crystals is reduced as spherical and finely dispersed particles are obtained in the aluminum matrix. The changes in size and morphology of the discontinuous silicon phase are significant since they have a direct influence on the mechanical properties.

A number of researchers have proposed [25,26,27] that the spheroidization process of silicon through solution heat treatment takes places in two stages: dissolution/separation of the eutectic branches and spheroidization of the separated branches. In the first stage, the Si particles are separated into segments at the corners of thin growth steps but retain their flake-like morphology. In the second stage, the broken segments spheroidize, and the aspect ratio decreases. The dissolution stage has the greatest effect on the time required to complete spheroidization and is strongly affected by the morphology of the Si particles; the smaller the flake length, the greater the spheroidization [24,28]. 

Any process which promotes eutectic branching, whether modification or higher cooling rate, will speed up the progress of separation and spheroidization. Modification by addition of impurities tends to refine the eutectic Si greatly, to promote twin branching, to raise the energy state with its inhomogeneity and consequently to promote the kinetics of the granulation of eutectic silicon. It was observed that after solution treatment of the RM and RGM alloys, the average Si particle area, length and roundness increased, while the aspect ratio and density decreased, compared to the as-cast condition, which may also be seen in Figure 5b and Figure 6b. 

In the absence of a modifier, the density of the Si particles was seen to increase by about 43% (from 10,096 to 14,457 particles/mm^2^) after solution heat treatment. Due to the spheroidization process described previously, fragmentation of the eutectic Si takes place, and spheroidization of the separated branches begins. The rate of spheroidization is affected by the segment size; subsequently, the smaller particles eventually spheroidize and coarsen while other large ones continue to segment, thereby accounting for the smaller variations in particle density as obtained for the unmodified alloy compared to the modified specimens.

**Figure 7:**
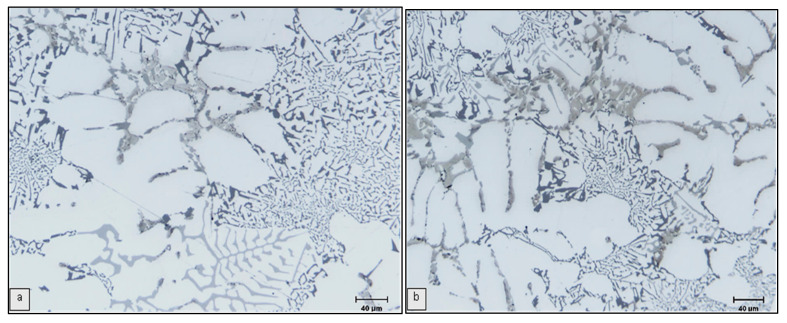
Optical micrographs showing the effects of Cu and Mg addition on Si morphology in as-cast (**a**) RC3 and (**b**) RC5 alloys.

In the as-cast condition, the average Si particle length increased from 2.69 to 3.11 and 5.16 µm for alloys RGM, RC2 and RC5, respectively, i.e., by 16% and 92% in the two RC alloys, while the average particle area increased (from 2.67 to 3.41 and 7.46 µm^2^, respectively) for the same alloys. The observed increase in the Si particle average length/surface area may be interpreted in terms of the interaction between Mg and Cu with the Si and Sr leading to the formation of Mg_2_Sr(Si,Al) and Al-Cu-Sr compounds, respectively. As a result, there is a depletion in the Sr concentration that is needed to obtain a fully modified alloy. Figure 6 shows that increasing the amounts of added Mg and Cu significantly reduces the modification effect of Sr in RGM alloy. The microstructures of RC3 and RC5 alloys show that some Si areas are fully modified, whereas other areas are only partially modified as depicted in Figure 7. 

After solution heat treatment for 8 h at 495 °C, the Si particle characteristics remained more or less unchanged compared to the as-cast condition. From this it may be concluded that increasing the levels of Mg and Cu hinders the effect of solution heat treatment on the Si particles. In this context, it is worth noting that the R alloy exhibited the highest average values for particle area and length, whereas the RM alloy showed the lowest of all the alloys investigated. This implies that any further addition of alloying elements would decrease or weaken the effect of Sr as a modifier as a result of their interaction with Sr to form complex intermetallic compounds.

### 3.2. Iron-Rich Intermetallics

In addition to the silicon structure, another important consideration from the point of view of microstructure is controlling the Fe content of the alloy. Accurate identification of the relatively coarse Fe-rich intermetallic phases commonly found in Al-Si casting alloys is also important, since some of these phases are associated with reduced mechanical properties. Figure 4b shows the microstructure of the R alloy in which the α-Fe phase appears in the form of small Chinese-script particles interspersed with Si particles; this observation indicates that the α-Fe particles had precipitated in co-eutectic or post-eutectic reactions. When 150 ppm Sr is added to the base alloy (i.e., the RM and RGM alloys), the α-Fe phase precipitates in the form of a pre-dendritic phase, as shown in Figure 5b and Figure 6b. 

**Figure 8:**
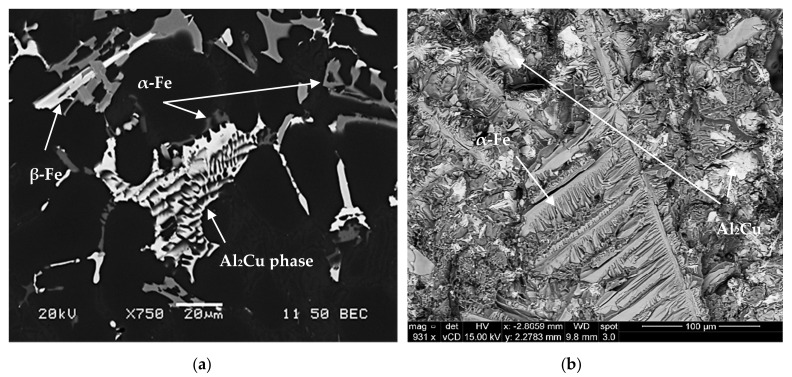

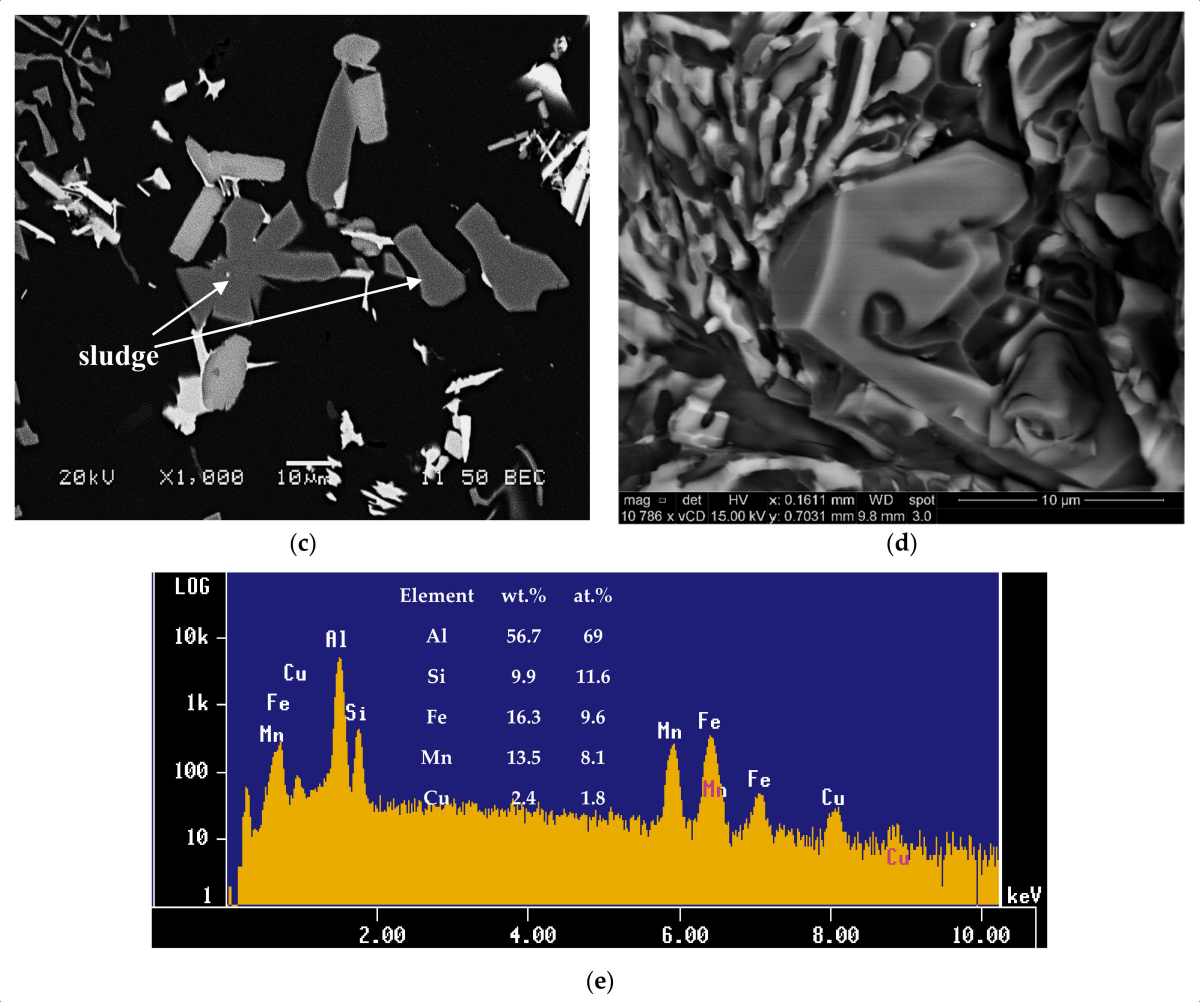
(**a**) Backscattered electron image showing co-existence of α-Fe, β-Fe and Al_2_Cu phases, and (**b**) fracture surface of as-cast tensile test bar showing the branching of α-Fe in three directions, RF4 alloy, (**c**) sludge particles in RF2 alloy, (**d**) deeply etched micrograph of sample of RGM alloy solutionized at 495 °C/8 h, revealing the persistence of α-Fe, (**e**) EDS spectrum corresponding to (**b**).

The backscattered images shown in Figure 8a,b reveal the influence of iron concentrations, at each level of manganese, on the formation of different iron intermetallics. It is seen that when the Fe content increases from ~0.5 wt.% (RGM alloy) to 1 wt.% (RF4 alloy), platelet-like β-Fe and Chinese-script α-Fe compounds form at low levels of manganese content. Upon increasing the manganese level (RF2 alloy), the Al(Fe,Mn)Si primary particles of sludge which precipitate directly from the liquid display a predominantly polyhedral shape; they are located within the α-Al dendrites, as shown in Figure 8c, where the clear, sharp edges of the particles confirm that they have not been transformed into the α-script, as was reported elsewhere [6,7]. 

**Figure 9:**
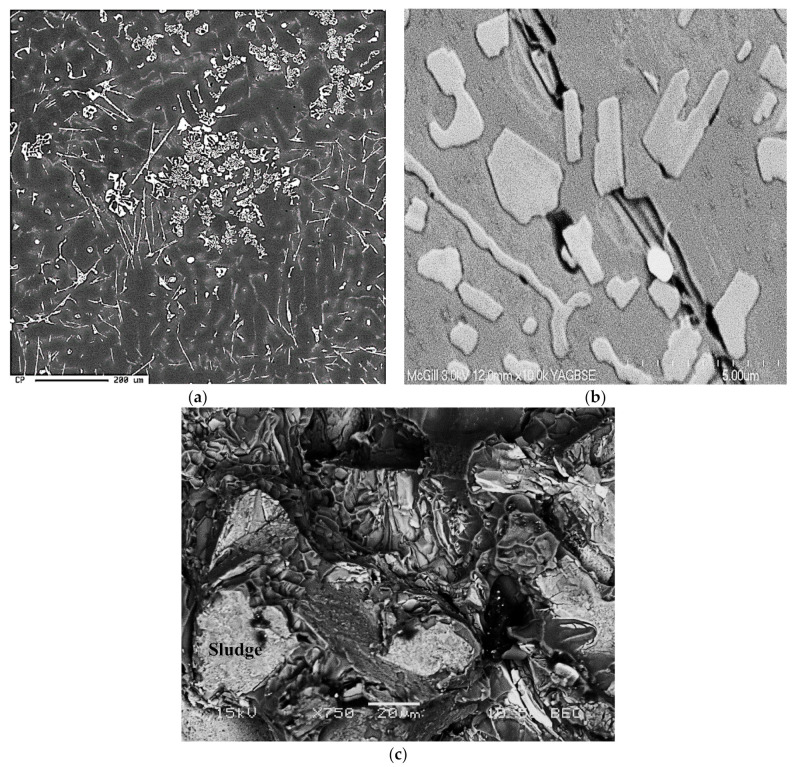
(**a**) Backscattered electron image showing precipitation of sludge particles along with α-Fe and β-Fe phase particles, (**b**) BSE image revealing the morphology of sludge, (**c**) fracture surface of the corresponding tensile bar-in alloy RF2.

The presence of hard sludge particles within the soft α-Al dendrites should lead to a more uniform distribution of the stresses throughout the alloy matrix and, hence, to improved mechanical properties. This shows that the precipitation of sludge particles need not necessarily be harmful to the alloy, as is commonly perceived in the literature, where the sludge particles are usually observed in the interdendritic regions. This phenomenon of iron intermetallic precipitation within the α-Al dendrites proves very useful in the case of such Al-Si die-casting alloys as 380 alloy, containing 9% Si, where the proportion of α-Al dendrites is relatively higher [29]. The various phases shown in Figure 9 were analyzed using wavelength dispersion spectroscopy (WDS), and identified from the corresponding chemical compositions. 

**Figure 10:**
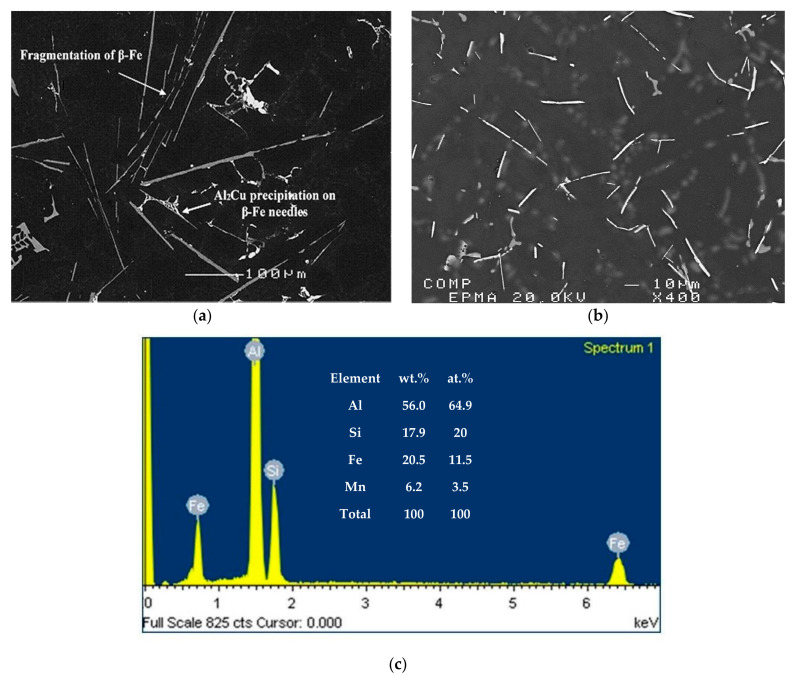
Backscattered images of Fe intermetallics observed in alloy RF4 (**a**) in the as-cast condition and (**b**) after solution heat treatment. (**c**) EDS spectrum corresponding to (**a**).

The polyhedral intermetallic compounds i.e. sludge have the same chemical composition as the α-Fe phase, and they remain unaffected by solution heat treatment, as shown in Figure 9. On the other hand, as shown in Figure 10, fragmentation of β-Fe due to the modification effect of Sr was observed, leading to a breakdown of the β platelets into small thin fragments by means of two mechanisms: (i) splitting of the needle into two halves through the formation of longitudinal cracks and (ii) fragmentation through Si rejection. These results are consistent with the research of Tahiri et al. [16,17], who reported that Sr has a poisoning effect on the nucleation sites for β-Fe platelets. The partial dissolution of β platelets becomes more pronounced after solution heat treatment. These observations confirm the findings of Villeneuve and Samuel [25] on the fragmentation and dissolution of the β-Fe phase during solution heat treatment of Al-13%Si-Fe alloys at 540 °C.

**Figure 11:**
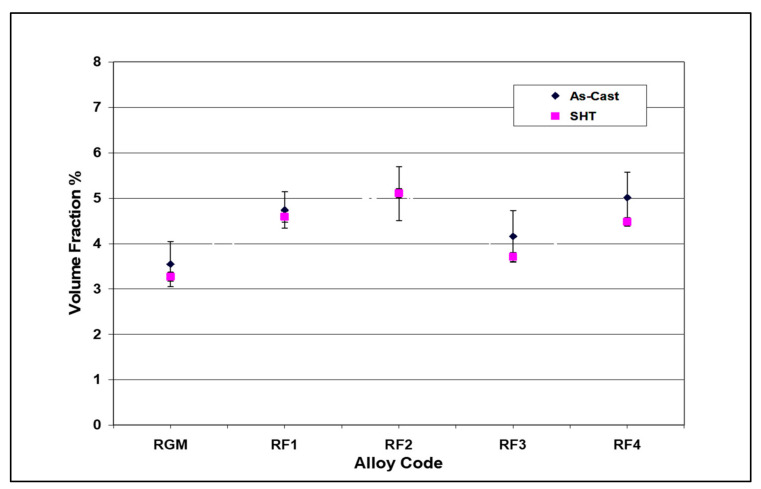
Volume fraction (%) of Fe intermetallics as a function of Fe and Mn addition to RGM alloy.

The volume fraction of iron intermetallics was plotted as a function of the amount of Fe and Mn added to the RGM alloy, as shown in Figure 11. It was found that this volume fraction increased with the addition of increasing levels of Fe and Mn. The volume fraction of iron phases after solution heat treatment came closer to their values in the as-cast condition for each Fe/Mn combination. These observations indicate that the α-Fe and sludge phases are not affected by solution heat treatment. On the other hand, due to the partial dissolution and fragmentation of the β-Fe phase during solution heat treatment, the volume fraction of iron phases decreased markedly at high levels of Fe and low levels of Mn (RF4 alloy) compared to the as-cast condition.

### 3.3. Copper-Rich Intermetallics

**Figure 12:**
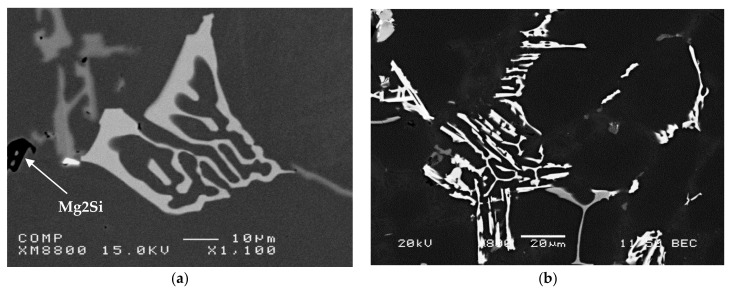

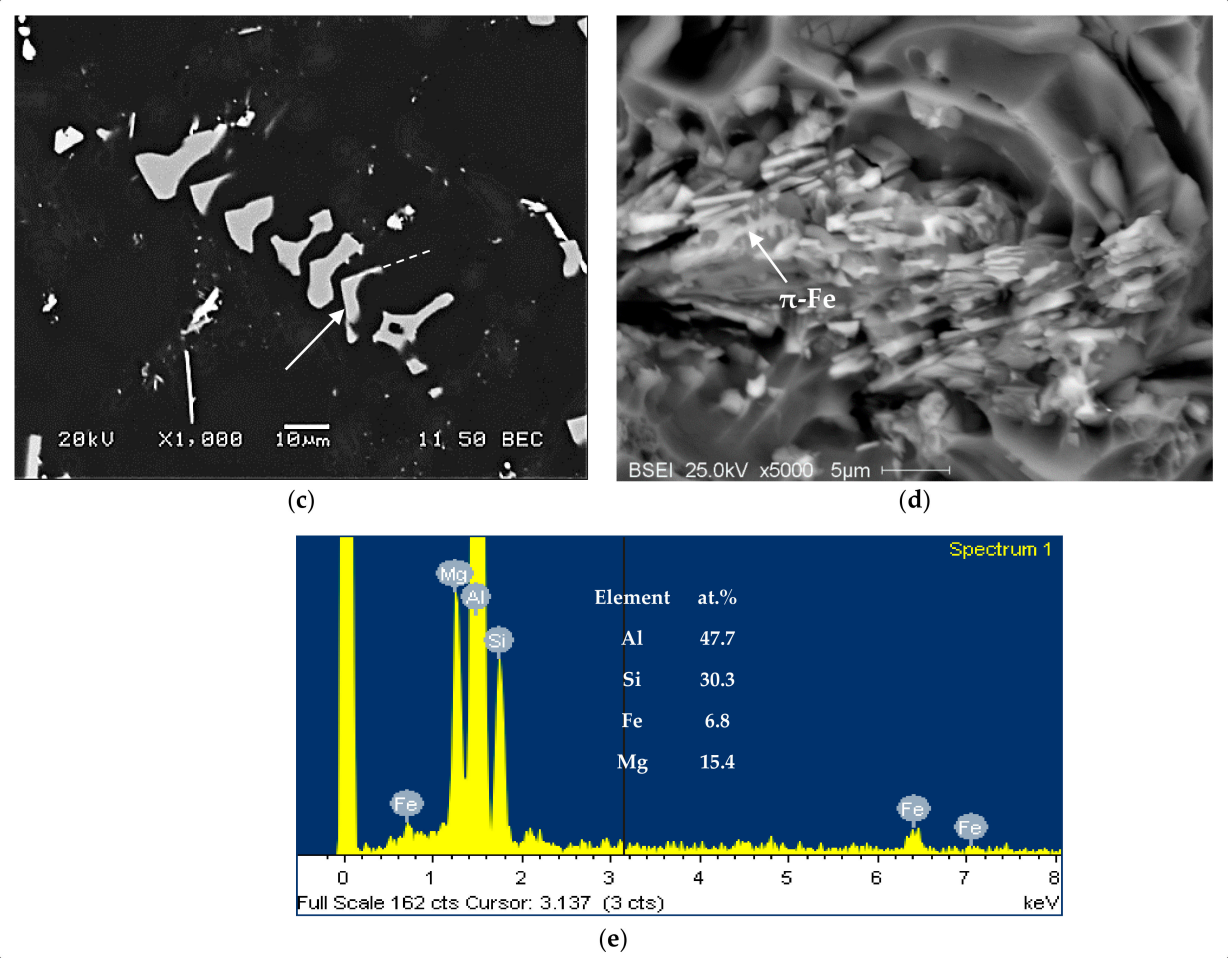
(**a**) Backscattered electron image of π-Fe, (**b**) decomposition of π-Fe into β-Fe during solutionizing treatment, (**c**) precipitation of the new β-platelets in the form of fishbone, (**d**) fracture surface of solutionized tensile bar revealing the presence of a packet of short β-Fe platelets corresponding to (**b**), alloy RC5. The white arrow in (**c**) points to the plate-like shape (broken line) of the new phase, whereas (**d**) shows traces of the original π-Fe phase; (**e**) EDS spectrum corresponding to (**a**).

As inferred from Figure 2, during the course of solidification, the β phase undergoes partial decomposition into a new π phase in the form of pale colored Chinese script as shown in Figure 12a, having a stoichiometric composition of Al_8_Mg_3_FeSi_6_, alloys RC1 through RC5. According to Elsharkawi et al. [30,31], this phase is unstable and decomposes into short platelets of β-Al_5_FeSi during SHT as illustrated in Figure 12b. Figure 12c shows precipitation of the new β-platelets in the form of fishbone structure; the broken line points to their plate-like nature. Traces of the original π phase (arrowed) together with the new β-platelets are clearly observed on the fracture surface of a tensile-tested sample of alloy RC5 in Figure 12d, while Figure 12e shows the corresponding EDS spectrum corresponding to the π phase in (a). 

**Figure 13:**
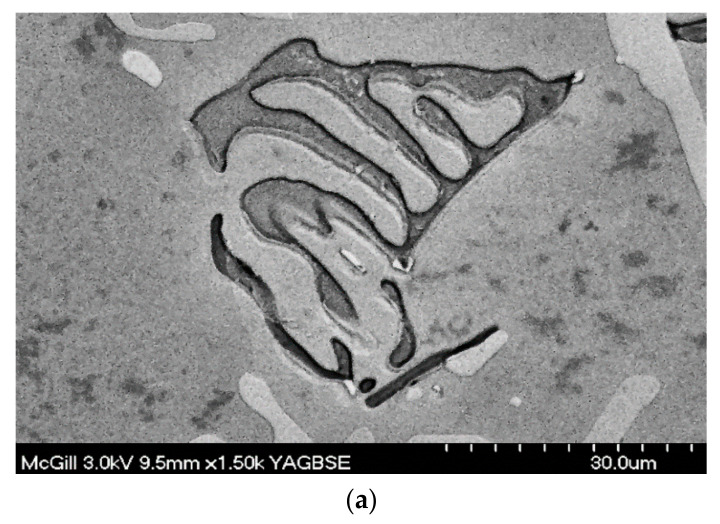

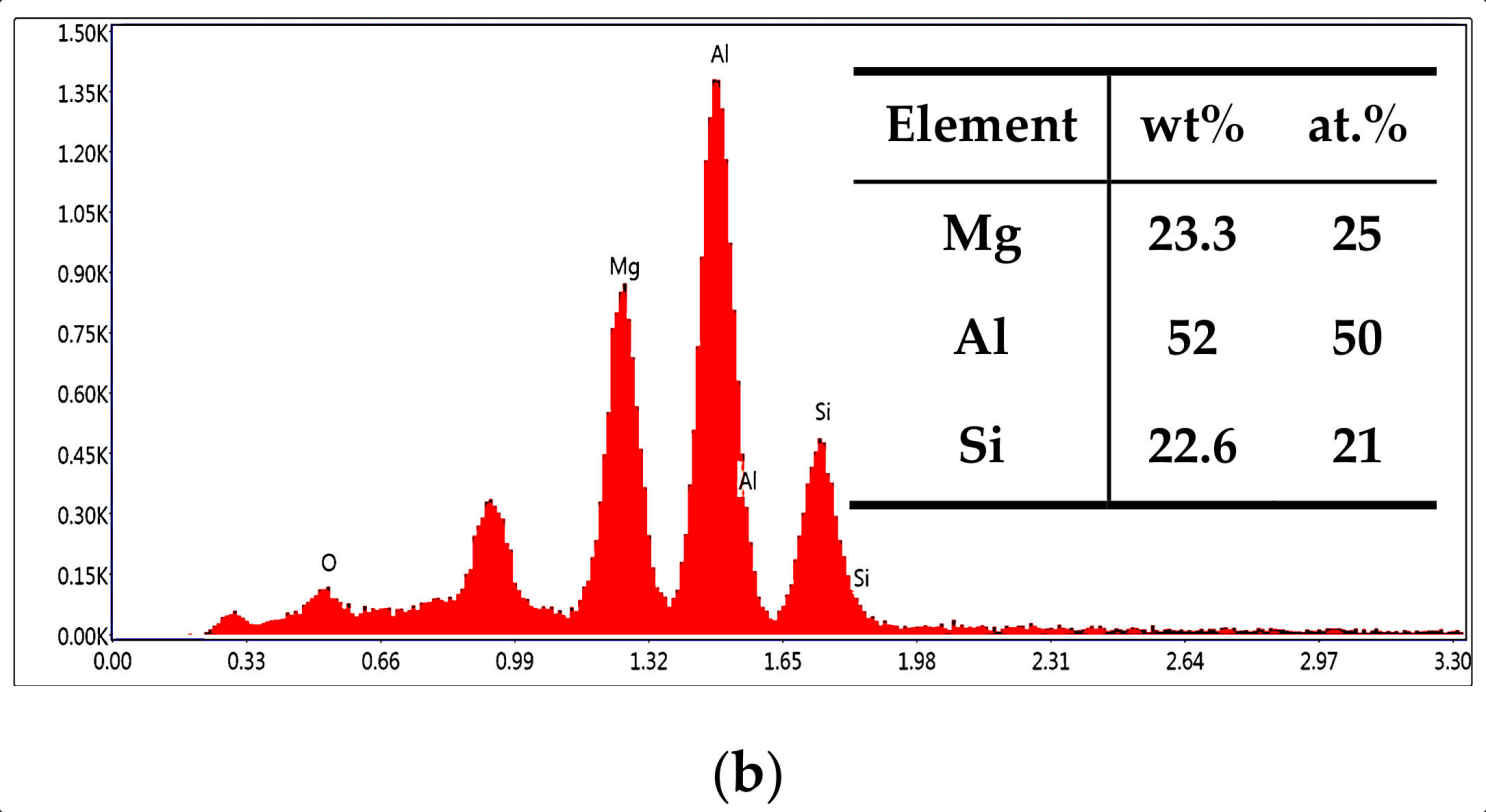
(**a**) Backscattered electron image of Mg_2_Si observed in Figure 12a, (**b**) corresponding EDS spectrum, alloy RC5.

Figure 13a displays a high magnification image of the Mg_2_Si phase observed in alloy RC5, with the corresponding EDS spectrum shown in Figure 13b. The presence of this phase may also be noted in Figure 12a, as the black script particle labeled Mg_2_Si. It precipitates in accordance with the solidification curve shown in Figure 2. 

Copper forms the intermetallic phase Al_2_Cu with aluminum which precipitates during solidification. Depending on the cooling rate and the local concentration of segregated Cu atoms, Al_2_Cu may precipitate in a block-like form directly from the liquid at a Cu concentration of ~53.5 wt.%, especially in the presence of the β-Fe phase, in the form of eutectic (Al + Al_2_Cu) or, as in many cases, as a mixture of both. 

**Figure 14:**
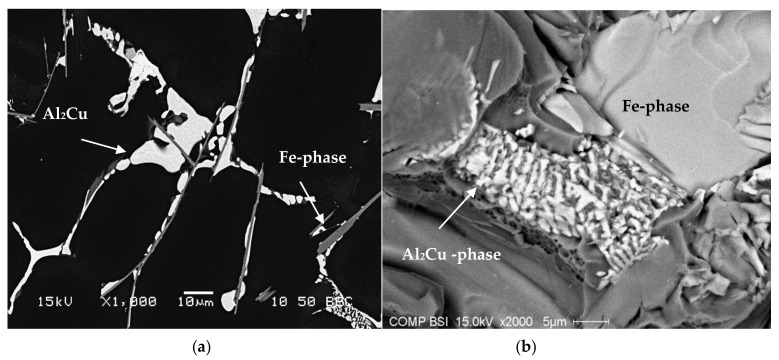

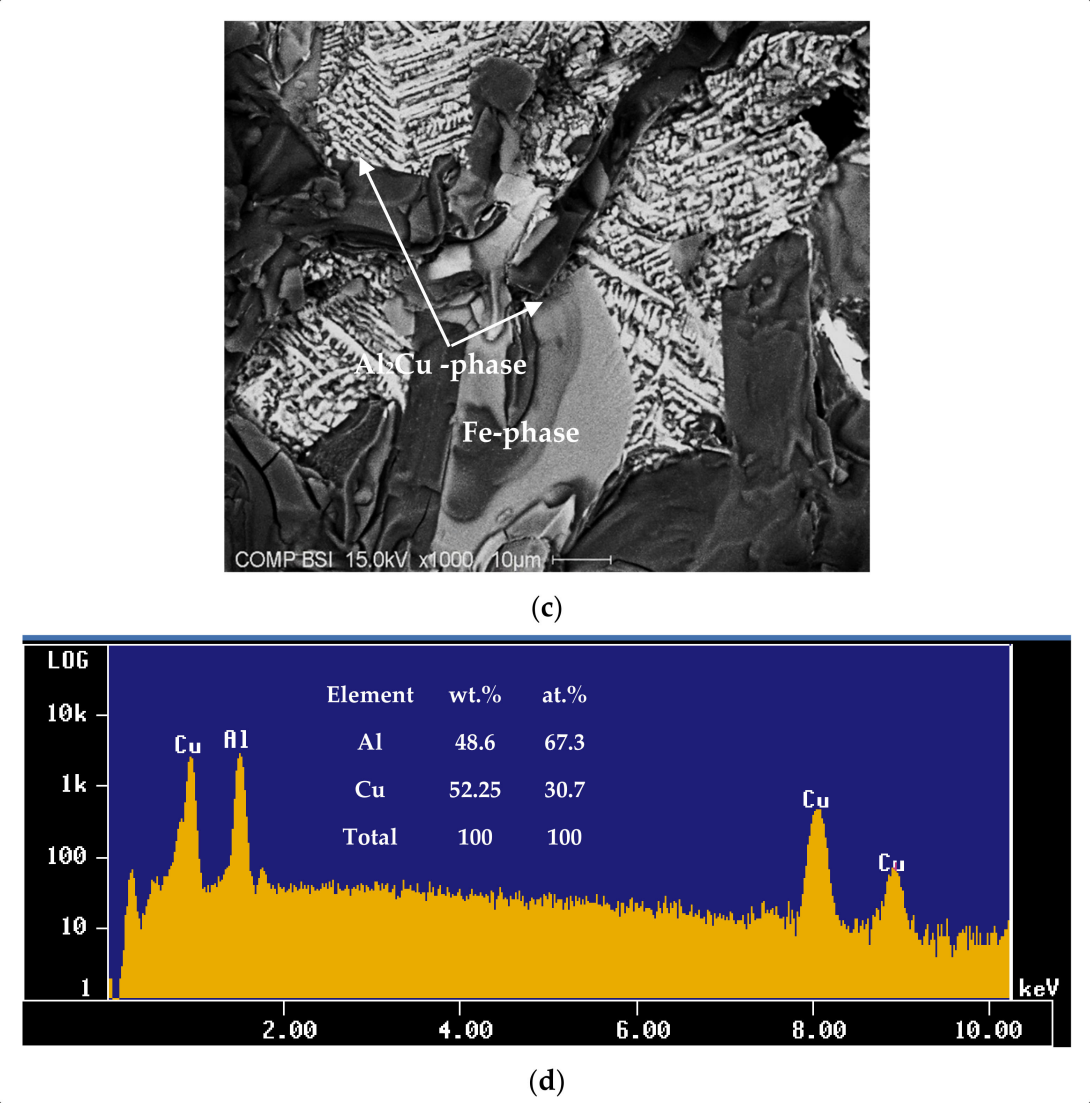
(**a**) Backscattered electron image showing precipitation of Al_2_Cu phase particles on the surfaces of β-Fe platelets, (**b**) the fracture surface of tensile bar of as-cast RF4 alloy, (**c**) segregation of Al_2_Cu phase in the form of towers in Sr-modified R alloy, alloy RGM, (**d**) EDS spectrum corresponding to (**c**).

Figure 14a shows the as-cast microstructure of the base R alloy, in which the copper phase is seen mainly as small pockets of the blocky Al_2_Cu phase nucleating on pre-existing β-Fe platelets. The backscattered image of the as-cast RF4 alloy (with ~1.0% Fe and 0.5% Mn), as seen in Figure 14b, shows that the β-Fe platelets and blocky Al_2_Cu particles are connected to each other, indicating that the β-Fe platelets act as nucleation sites for the copper phase particles. Segregation of the Al_2_Cu phase is clearly noted on the fracture surface of the Sr-modified RM and RGM alloys, an example of which is shown in Figure 14c, while Figure 14d shows the corresponding EDS spectrum. A similar observation was reported by Samuel et al. [32] in 319-type alloys.

**Figure 15:**
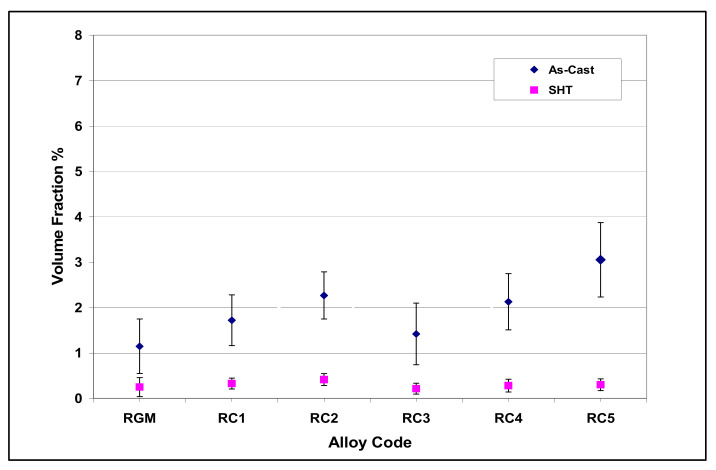
Volume fraction (%) of undissolved Cu intermetallics as a function of Cu and Mg addition to RGM alloy.

**Figure 16:**
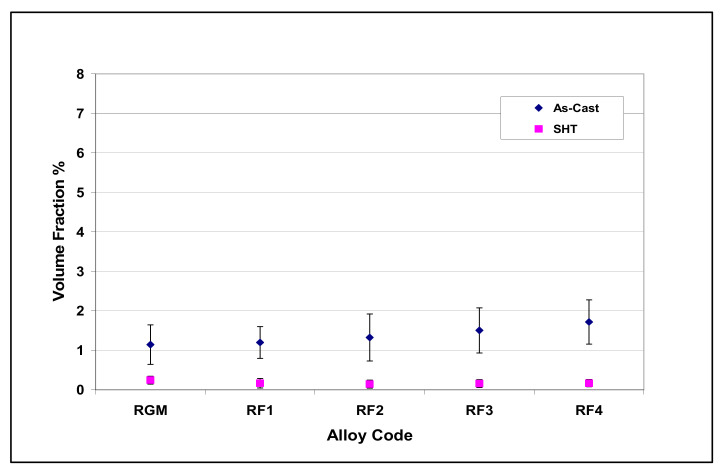
Volume fraction (%) of undissolved Cu intermetallics as a function of Fe and Mn addition to RGM alloy.

The addition of Sr to the R alloy (RM and RGM alloys) leads to the segregation of the Al_2_Cu particles in regions away from the growing Al-Si eutectic colonies, as shown in Figure 14c. In order to study the effects of solution heat treatment, the volume fraction of the undissolved copper phase was measured for the various alloy samples/conditions. The amounts of the undissolved copper phase thus determined were plotted as a function of different alloying elements added to the RGM alloy and are presented in Figure 15 and Figure 16. It can be observed that the quantity of the Al_2_Cu phase after solution heat treatment decreased abruptly for all alloys compared to other cases in the as-cast condition. About 78% of the total Al_2_Cu phase was dissolved in the matrix of the RGM alloy; this observation was confirmed by subsequent examination of the microstructure.

The highest dissolution of the Al_2_Cu phase was observed in the solution-treated RF4 and RC5 alloys (90% in each case). As regards the RF4 alloy, the high level of iron (~1%) assisted in the dispersion of the Al_2_Cu particles and the formation of the β-Fe phase which precipitated prior to the Cu-rich phase, thereby providing nucleation sites for the Al_2_Cu particles, while also reducing both Al_2_Cu phase segregation and, hence, the size of the Al_2_Cu particles. Thus, during solution heat treatment, these Al_2_Cu particles would be much more easily dissolved as a result of the reduction in their size. The high degree of dissolution of the Al_2_Cu phase in the RC5 alloy may be attributed to the presence of Mg (0.5 wt.%) which lowers the temperature of eutectic Si and, consequently, that of all the subsequent reactions, leading to faster dissolution of Al_2_Cu particles during solution heat treatment. 

**Figure 17:**
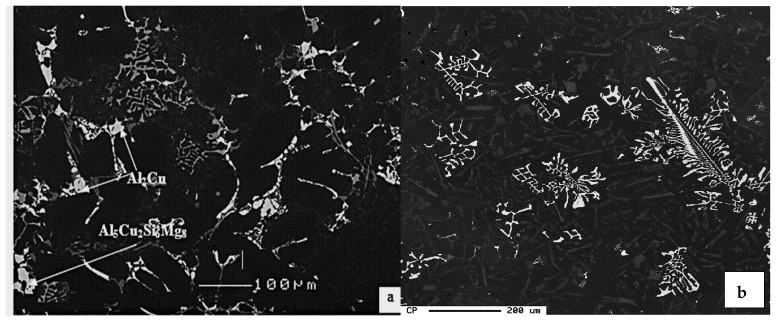
Backscattered images showing Al_2_Cu and AlCuMgSi phase particles observed in: (**a**) as-cast and (**b**) solution-treated RC3 alloy.

At the same time, it was also observed that during the process of dissolution, the Al and Cu concentrations in the Al_2_Cu phase remain virtually stable. It may be concluded, therefore, that the dissolution of the Al_2_Cu phase occurs by diffusion, into the surrounding matrix, of the Cu atoms located in the outer layer of the Al_2_Cu phase particles, without changing the chemical composition of the remaining portion of the particles. Figure 17a shows a backscattered micrograph obtained of the RC3 alloy in the as-cast condition, whereas Figure 17b reveals almost complete dissolution of the phase after solution heat treatment for 8 h at 495 °C. The white spots in Figure 17b point to traces of the undissolved Al_2_Cu phase. 

**Figure 18:**
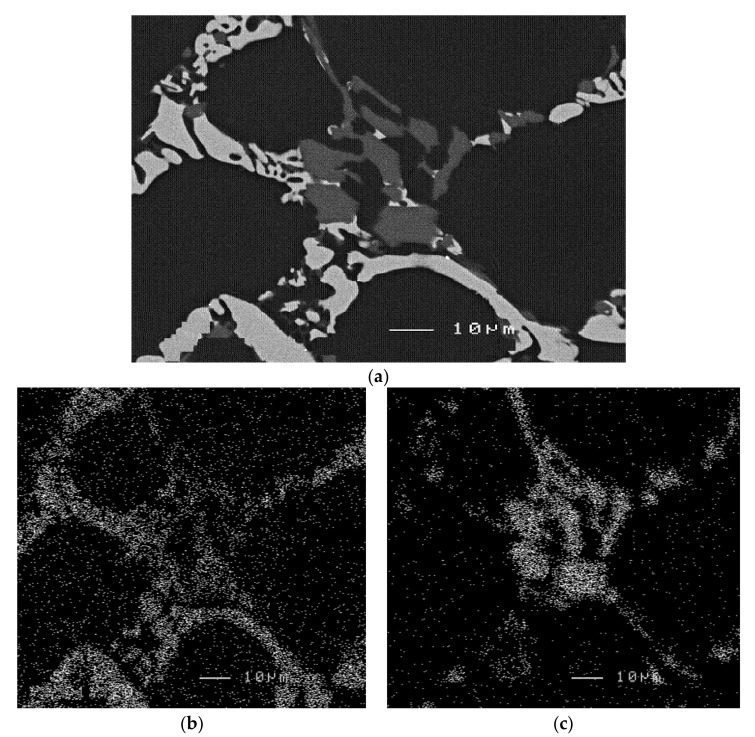
(**a**) High magnification backscattered image taken of RGM alloy in as-cast condition showing Al_2_Cu and AlCuMgSi phase particles, X-ray images of (**b**) Cu, and (**c**) Mg concentration, corresponding to the backscattered image shown in Figure 18a.

In Al-Si-Cu-Mg systems, precipitation of Al_5_Cu_2_Mg_8_Si_6_ is reported to take place at the end of the Al-Al_2_Cu eutectic reaction [33]. In most cases, this phase appears in the form of small grey particles growing out of the Al_2_Cu phase particle clusters as shown in Figure 18a, which represents a high magnification backscattered image of the RGM alloy. The distribution of Cu- and Mg-containing particles is shown in Figure 18b,c, respectively. The amount of the phase is found to increase progressively with an increase in Mg content. It is interesting to observe the persistence of this phase after solution heat treatment, as shown by the WDS analysis of the phase provided in Table 3. It should be mentioned here that the WDS analysis of these particles revealed an unexpectedly higher concentration of aluminum, by 3 wt.%, than that obtained for the as-cast condition. This observation may be an indirect indication of the sluggish dissolution of the Al_5_Cu_2_Mg_8_Si_6_ phase during solution heat treatment. 

### 3.4. Porosity 

**Figure 19:**
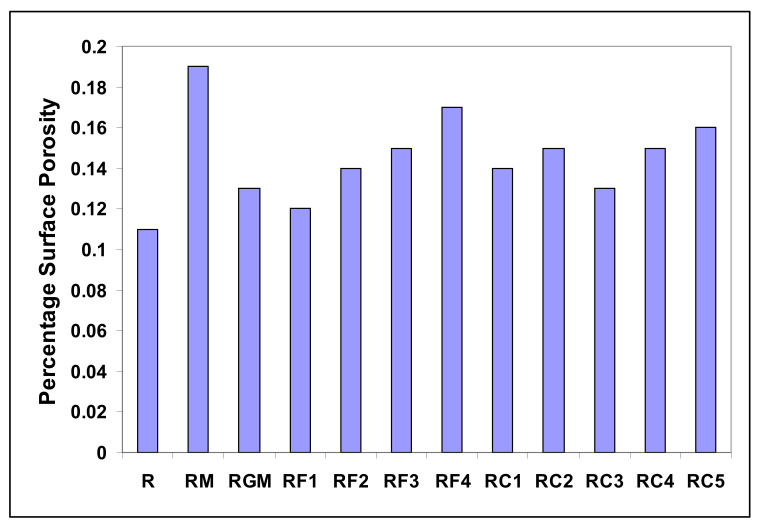
Percentage surface porosity as a function of the addition of alloying elements in various as-cast alloys prepared from the experimental R alloy.

There are several factors which affect porosity formation in Al-Si alloys. Alloying is a factor which can seriously complicate the study of porosity since alloying influences almost every aspect of the solidification process as well as a variety of metallurgical, physical and chemical properties. Changes from one alloy to another may cause considerable confusion in the study of porosity since the results are often not comparable. For the purposes of the current study, porosity characteristics were analyzed and quantified using a Clemex image analyzer in conjunction with an optical microscope. Figure 19 and Table 4 provide pertinent data for various alloy samples in the as-cast condition with regard to the percentage surface porosity, which may be defined as the area fraction of porosity observed on a measured sample surface area. It should be noted here that in all of the cases, the liquid metal was continuously degassed prior to casting in order to minimize the effects of gas- and inclusion-related porosity. 

Modifying alloy RM with Sr resulted in more porosity than that observed in the same alloy before modification. As described by Campbell [34] the pore growth rate can be expressed as:

Pg + Ps > Patm + PH + Ps-t

where Pg = equilibrium pressure;

Ps = drop pressure;
Patm = atmospheric pressure;
PH = metallostatic pressure;
Ps-t = surface tension pressure.
Pg and Ps are considered as the major driving forces in the formation of porosity.

Therefore, the increase in amount of porosity in the Sr-modified alloy, i.e., RM alloy, may be interpreted in terms of an increased pore size and density. It is inferred from the work of Samuel et al. [35] that increasing the pore size is more harmful to the alloy’s mechanical properties, which is another parameter to be considered in the use of grain refining, which may lead to a reduction in the porosity, as the application of grain refining is reported to be effective in reducing the pore size [36]. 

As shown in Table 4, precipitation of the α-Chinese-script phase when Mn was added to the melt, i.e., RF1 or RF2 alloy, results in the formation of fine pores. The shape of the α-Fe phase (see Figure 8) enhances the passage of the liquid metal between the dendrite arms of the particles. In contrast, the β phase platelets could block the filling between the platelets causing the formation of large pore sizes. Table 4 shows that the Mn-containing alloys display porosity values comparable to those obtained from the base 396 (R) alloy.

**Figure 20:**
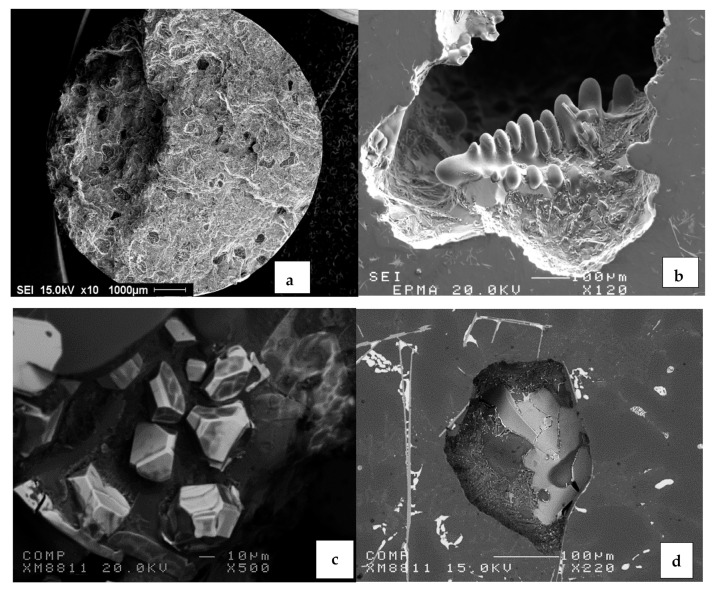

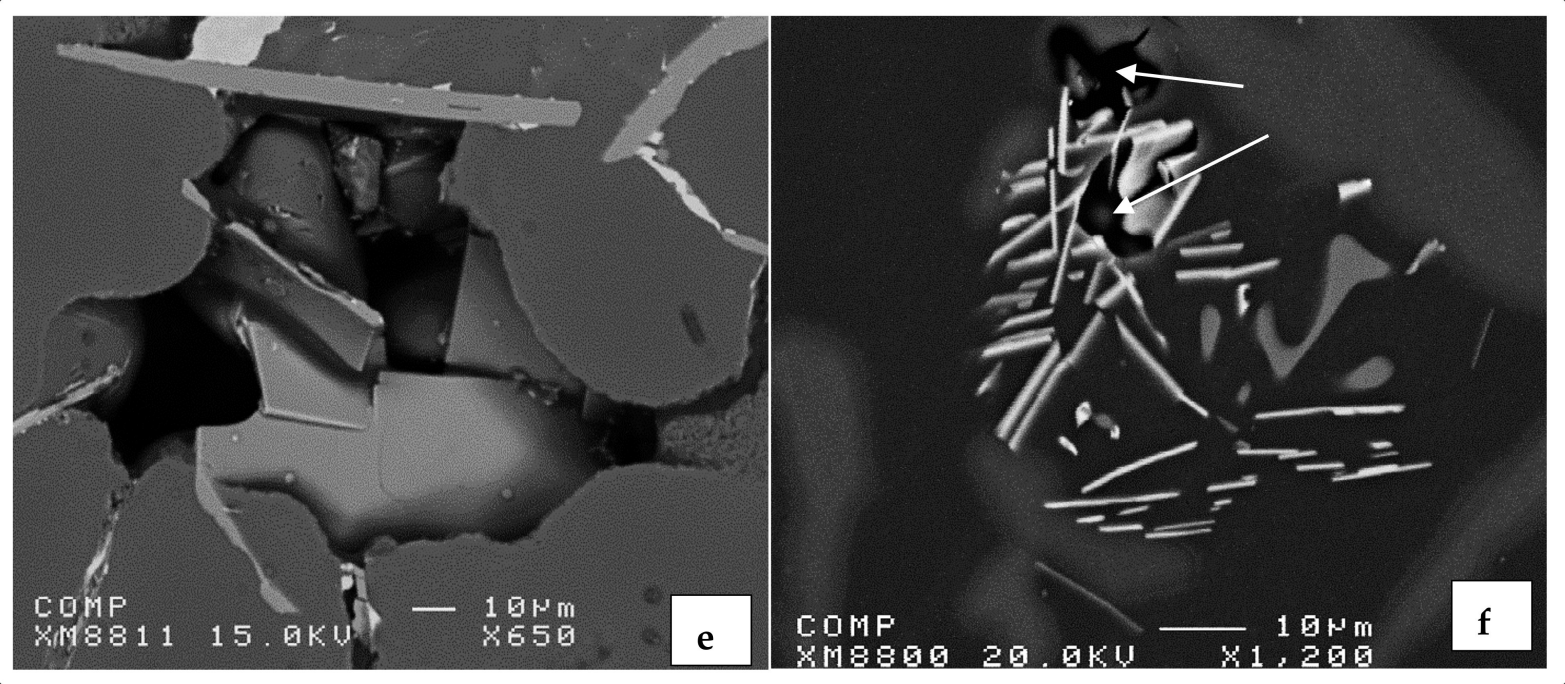
Examples of porosity observed in the present alloys: (**a**,**f**) gas porosity, no degassing. (**b**) shrinkage porosity, (**c**) porosity formed due to over-modification, (**d**) gas porosity nucleated on the surface of β-platelets, (**e**) shrinkage porosity formed by intersection of thick β-platelets, (**f**) micro-porosity (arrowed) formed by a packet of small β-platelets (see Figure 10).

It has been reported by Edwards et al. [27] that dispersed microporosity may occur in alloys with Cu as an alloying element (almost four times that of Cu-free alloys). The authors claim that this observation is due to the Liq → α-Al + Si + CuAl_2_ reaction at about 525 °C. As a result, regions containing ternary liquid may solidify during the casting process, making them difficult to feed. It can be observed that porosity in the RC group of alloys is barely affected by the addition of Cu and Mg, as pointed out by Edwards et al. [27]. Figure 20 displays some examples of porosity observed in the present alloys.

### 3.5. Hardness

**Figure 21:**
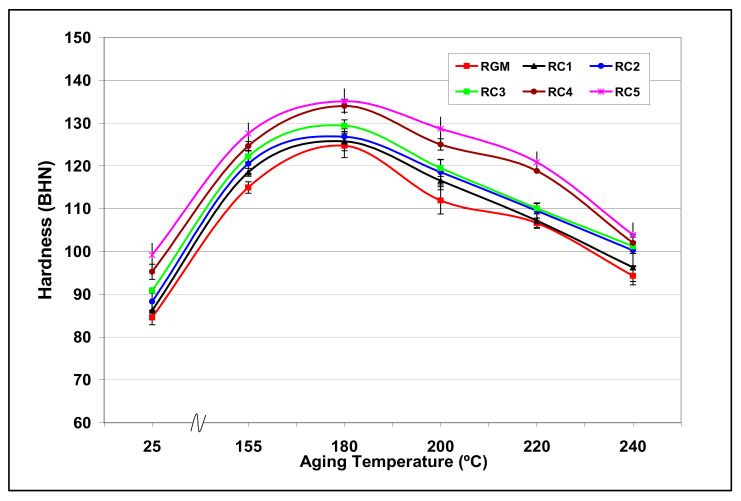
Variation in Brinell hardness values (BHN) in RCM and RC alloys as a function of heat-treatment conditions.

Figure 21 shows the combined effects of Cu and Mg content with aging temperature on the RGM alloy. It is observed that the addition of Cu and Mg has the effect of improving the peak-aging hardness of the RGM alloy. For each alloy in the RC group, the hardness first increases with an increase in aging temperature of up to 180 °C, decreasing thereafter as the aging temperature increases further. The increase in hardness with the addition of Cu and Mg may be attributed to the formation of the hard and brittle (metastable) intermetallic phases Al_2_Cu and Al_2_CuMg and to an increased bonding of the silicon particles with the matrix, where the thermal energy is sufficient to precipitate such intermediate phases as are coherent with the matrix. The decrease in hardness at aging temperatures above 180 °C may be attributed to the coarsening of the various microconstituents and to a decrease in cohesion with the matrix. The coarsening of hard intermetallic phases may reduce the barrier to dislocation movement and, hence, to flow stress/hardness. 

The effect of increasing the Mg content to 0.5% at different levels of Cu produces higher hardness values than those obtained for alloys containing 0.3% Mg, indicating that hardening which results from Al_2_CuMg precipitation adds to that achieved due to Al_2_Cu precipitation. This is also evident from a comparison of the amounts of Cu and Mg present in the two alloys: ~2.25% Cu and ~0.5% Mg in the RC3 alloy vs. ~3.25% Cu and ~0.3% Mg in the RC2 alloy. In the RC5 alloy, which contains ~3.25 wt.% Cu and ~0.5 wt.% Mg, the peak hardness value is seen to increase slightly by about 10% at 180 °C compared to the RGM alloy. This may be interpreted in terms of the formation of complex insoluble phases, such as Al_5_Mg_8_Si_6_Cu_2_, which decrease the amount of free Mg and Cu available for further hardening during the aging process.

## 4. Conclusions

1. The addition of Sr leads to the segregation of the copper phase in the form of towers made of several layers of Al-Al_2_Cu in areas away from the modified eutectic Si; it also alters the precipitation of the α-Al_12_(Fe,Mn)_3_Si_2_ phase from a post-dendritic reaction in the unmodified alloy to a pre-dendritic one in the modified alloy. This observation is consistent with the published data.
2. An increase in the level of Mg and Cu supplied to the Sr-containing alloys results in an increase in the Si particle size and reduces the roundness ratio and particle density, thereby, in effect, diminishing the modifying influence of Sr. The addition of Fe and/or Mn, however, has no significant effect on the Si particle characteristics.
3. As the Fe content increases, more intermetallic compounds form at each level of Mn, and the volume percent of Fe-intermetallics increases. The stoichiometry of polyhedral (sludge) and Chinese-script (α-Fe) intermetallics corresponds to Al_12_(Fe,Mn)_3_Si_2_, while platelet compounds (β-Fe) have the stoichiometry Al_5_(Fe,Mn)Si and the π-Al_8_Mg_3_FeSi_6_ phase. The Al_5_Cu_2_Mg_8_Si_6,_ α-Fe, and sludge intermetallics are insoluble, whereas the platelet-like β-Fe phase dissolves partially during solution heat treatment at 495 °C/8 h.
4. The percentage surface porosity increases significantly with Sr addition. This observation is consistent with the published data. The effects of Cu, Mg and Mn are less clear. It appears that they have no measurable effect on porosity at the levels used in this study.
5. Over-modification may, however, have an effect on porosity formation caused by precipitation of primary Al-Si-Sr phase particles.

## Figures and Tables

**Table 1:** Chemical analysis of the alloys used in the present study.

Alloy	Elements (wt.%)									
	Si	Cu	Mg	Fe	Mn	Sr	Ti	Al	Mn/Fe	SF ^b^
R ^a^*	10.89	2.243	0.309	0.464	0.492	0.014	0.057	bal.	1.069	1.06
RM	10.93	2.221	0.370	0.449	0.494	**0.030**	0.077	bal.	1.099	1.44
RGM	10.92	2.138	0.373	0.429	0.471	**0.030**	**0.22**	bal.	1.096	1.37
RF1	10.82	2.099	0.276	**0.733**	**0.667**	0.03	0.12	bal.	0.909	2.07
RF2	10.87	2.092	0.325	**0.848**	**0.801**	0.03	0.10	bal.	0.944	2.45
RF3	10.90	2.132	0.275	**0.735**	0.481	0.03	0.16	bal.	0.654	1.69
RF4	10.93	2.128	0.333	**0.980**	0.482	0.03	0.16	bal.	0.492	1.95
RC1	10.95	**2.726**	0.276	0.464	0.487	0.03	0.17	bal.	1.050	1.44
RC2	11.11	**3.308**	0.353	0.499	0.476	0.03	0.16	bal.	0.955	1.46
RC3	10.85	2.300	**0.491**	0.462	0.46	0.03	0.19	bal.	0.955	1.39
RC4	10.99	**2.743**	**0.552**	0.466	0.458	0.03	0.18	bal.	0.983	1.39
RC5	11.30	**3.128**	**0.604**	0.473	0.451	0.03	0.19	bal.	0.952	1.39

Bold numbers represent additives; ^a^*: Al-11%Si-2%Cu-Mg base alloy; ^b^: sludge factor, SF = (1 × wt.% Fe) + (2 × wt.% Mn) + (3 × wt.% Cr).

**Table 2:** Suggested main reactions occurring during solidification of alloy R [10].

Reaction	Suggested Temperature Range (°C)	Suggested Precipitated Phase
Al	600–597	- Formation of α-Al dendritic network
2. Al–Si, β–Fe and α–Fe	560–558	- Precipitation of Al-Si eutectic phase - Precipitation of post-eutectic β-Al_5_FeSi phase - In case of presence of Mn, precipitation of α-Al_15_(Mn,Fe)_3_Si_2_ phase
3. π–phase	525–523	- Transformation of β-Al_5_FeSi phase to π-Al_8_Mg_3_FeSi_6_ phase
4. Mg–Si	540–538	- Precipitation of Mg_2_Si phase
5. 5Al–Cu	500–496	- Formation of eutectic Al-Al_2_Cu phase
6. Q–phase	485–489	- Precipitation of Q-Al_5_Mg_8_Cu_2_Si_6_ phase

**Table 3:** Chemical analysis of phases observed in Figure 17.

Phase	Element	Wt.%	at.%	Approximate Formula
Cu phase (As-cast)	Al	48.57	67.3	Al_2.18_Cu
	Cu	52.25	30.74	
	Total	100.8	98.04	
AlFeMgSi π phase (As-cast)	Al		48.3	Al_7_._8_Fe_0.99_Mg_3_Si_4.44_
	Si		27	
	Fe		5.9	
	Mg		18.6	
	Total		99.54	
AlCuMgSi Q phase (solution-treated)	Al	16.61	17.72	Al_3.4_Cu_1.7_Si_5.8_Mn_8_
	Si	29.78	30.52	
	Cu	20.09	9.1	
	Mg	35.4	41.93	
	Total	101.8	99.27	

**Table 4:** Percentage surface porosity in samples obtained from various alloys.

Alloy Code	Addition	Surface Porosity (%)
R	No addition	0.11
RM	R alloy + 0.015% Sr	0.19
RGM	RM alloy + 0.25% Ti	0.13
RF1	RGM alloy + 0.25% Fe + 0.25% Mn	0.12
RF2	RGM alloy + 0.5% Fe + 0.5% Mn	0.14
RF3	RGM alloy + 0.25% Fe	0.15
RF4	RGM alloy + 0.5% Fe	0.17
RC1	RGM alloy + 0.5% Cu	0.14
RC2	RGM alloy + 1% Cu	0.15
RC3	RGM alloy + 0.5 Mg	0.13
RC4	RGM alloy + 0.5% Cu + 0.5 Mg	0.15
RC5	RGM alloy + 1% Cu + 0.5 Mg	0.16

## Data Availability

Data will be made available upon request.
